# Neurophysiological indicators of self-efficacy in internet gaming disorder: evidence from late positive potentials

**DOI:** 10.3389/fpubh.2026.1785973

**Published:** 2026-04-14

**Authors:** Hyunho Lee, Minkyung Park, Areum Choi, Ara Cho, So Young Yoo, Hong Choi, Jung-Seok Choi

**Affiliations:** 1 Department of Psychiatry, Samsung Medical Center, Sungkyunkwan University School of Medicine, Seoul, Republic of Korea; 2 Department of Psychiatry, SMG-SNU Boramae Medical Center, Seoul National University College of Medicine, Seoul, Republic of Korea

**Keywords:** craving, cue reactivity, ERP, Internet gaming disorder, self-efficacy

## Abstract

**Objective:**

This study investigated late positive potentials (LPP) and levels of self-efficacy and interpersonal relationships in internet gaming disorder (IGD), and examined their potential correlation to elucidate the relationship between neurophysiological and psychological aspects of IGD.

**Methods:**

A total of 91 participants were recruited, including 46 individuals with IGD (39 males, seven females; age range: 18–42 years) and 45 healthy controls (HCs; 31 males, 14 females; age range: 19–32 years). All participants completed a cue-reactivity task during electroencephalography (EEG) recording, in which game-related visual cues and neutral images were presented. Late positive potentials (LPP) were calculated as the mean amplitudes within the 400 and 700 ms time window at centro-parietal (CP3, CP1, CPz, CP2, and CP4) and parietal (P3, P1, Pz, P2, and P4) electrode sites. Participants also completed questionnaires including Generalized Self-Efficacy Scale (SES), and the Relationship Change Scale (RCS) for assessing self-efficacy and interpersonal relationships.

**Results:**

A significant Electrode × Group interaction was observed in the centro-parietal electrodes. Exploratory comparisons indicated nominal group differences in the mean LPP amplitudes at the CP3 and CP1 electrode sites; however, these effects did not survive correction for multiple comparisons. Significant negative correlations were found between SES score and LPP amplitudes across parietal electrode sites (P3, P1, Pz, P2 and P4). These correlations remained significant after adjustment for covariates (Beck Depression Inventory, Beck Anxiety Inventory) and correction for multiple comparisons.

**Conclusions:**

This study demonstrated that adults with IGD may exhibit an altered topographical pattern of LPP amplitudes in response to game-related cues in the centro-parietal regions. Importantly, LPP amplitudes in the parietal region were significantly associated with individual self-efficacy levels. These findings suggest that LPP amplitudes in the centro-parietal region may serve as a preliminary neurophysiological correlate reflecting the individual levels of self-efficacy in IGD.

## 1 Introduction

Since the advent of the internet, global internet usage has increased substantially. This trend has been accompanied by the emergence of internet gaming disorder (IGD), which is characterized by persistent and recurrent engagement in internet gaming activities (1). There is growing attention to IGD, which is reflected by its inclusion in the DSM-5-TR as a condition for further study and its formal recognition in the ICD-11 (1, 2). A recent meta-analysis reported that the pooled prevalence of IGD was 6.7% (3), which is comparable to that of other addictive disorders, including alcohol use disorder (4). Given its estimated prevalence and associated burden, it is necessary to enhance our understanding of IGD, including its neurophysiological mechanism.

As one of the key features of addictive disorders, the paradigm of cue-reactivity has been extensively investigated (5, 6). It has been proposed by several learning theories, which assume that people with substance addiction may develop conditioned reactions to stimuli (cues) associated with substance use (7). Although this concept was originally investigated in substance addiction, it is also being investigated in behavioral addictions (8). A meta-analysis demonstrated that people with behavioral addictions manifested heightened cue-reactivity in response to addiction-relevant stimuli compared to healthy controls, accompanied by increased neural activation in brain regions including the caudate nucleus, inferior frontal gyrus, median cingulate cortex, subgenual cingulate, and precentral gyrus (8). People with internet addiction or problematic internet use also seem to have similar features, showing more intense craving in response to addiction-relevant stimuli compared to non-addicts (9, 10).

For assessing cue-reactivity and attentional bias of addictive disorders, late positive potential (LPP) is often used in event-related potential (ERP) studies (11). LPP is a late, slow positive voltage change which typically begins 200–300 ms after the stimuli and reaches its maximum amplitude within 1 s (12). The peak amplitude is usually around 600 ms (13). It has been considered as a neural marker for attention to emotional stimuli, with larger LPP amplitudes reflecting greater emotional reactivity (14, 15). In substance use disorders, LPP has demonstrated its potential utility as a biomarker for motivational relevance (11). In IGD, the role of LPP is less investigated; however, our previous study reported significant differences in LPP amplitudes between individuals with IGD and healthy controls (16).

It is important to identify the risk and protective factors of IGD for the early intervention and prevention of the disease. Among them, the level of self-control and interpersonal relationship seems to play a role as a protective factor (17, 19). These aspects can be assessed in several domains, including self-efficacy (18) and interpersonal relationships (19). Self-efficacy refers to an individual's belief in one's capacity to execute behaviors necessary to produce specific achievements (20). Previous studies have reported reduced levels of self-efficacy and impaired interpersonal relationships in individuals with IGD (18, 19). Notably, there has been a report that the level of self-efficacy differed according to the frequency of internet gaming (21). The diminished level of self-efficacy in casual gamers who do not meet criteria for IGD implies that lowered self-efficacy may precede the development of IGD (21). These findings indicate that self-control-related constructs, particularly those related to self-efficacy, may serve as potential predictive factors for IGD.

At present, there are limited diagnostic tools for IGD, and the diagnostic criteria themselves have been criticized for insufficient reliability (3, 22). This limitation might be partly attributable to relatively short history of IGD and scarcity of data (23). Nevertheless, the prevalence of IGD continues to increase worldwide (24), suggesting that its clinical significance may be currently underestimated. Therefore, the identification of reliable and clinically meaningful markers is essential. Particularly, as with other psychiatric disorders, markers that bridge clinical characteristics and neurophysiological processes are valuable. Given that LPPs are closely associated with individual emotional stability (14, 15, 25), and that self-efficacy is linked to the emotional regulation (26), it is plausible that LPPs may be associated with individual levels of self-efficacy.

In IGD, this potential linkage may also be conceptualized in relation to established addictive pathways. Self-efficacy reflects one's capability to execute specific goal-directed behaviors (20) and is therefore inherently linked to motivational regulation. Cue-induced motivational urge in addictive behaviors is primarily related to the mesocorticolimbic dopaminergic pathway (27). However, accumulating evidence suggests that this neurophysiological process is modulated by regulatory control systems, which are closely related to executive function (28, 29). Therefore, lower self-efficacy may reflect reduced top-down regulatory modulation of motivation in response to addiction-related cues, resulting in higher LPP amplitudes. Although LPP represents a state-dependent neurophysiological response to emotionally salient stimuli, averaged LPP amplitudes from multiple trials can reflect stable individual differences in cue-reactivity. Thus, LPP amplitude may reflect individual differences in motivational and regulatory processes associated with trait-level constructs such as self-efficacy. From this perspective, investigating the association between LPP amplitudes and self-efficacy for IGD is warranted.

In the present study, we examined the late positive potentials and levels of self-efficacy and interpersonal relationships in individuals with IGD and healthy controls. Specifically, we assessed whether LPP amplitudes differed between IGD and healthy control groups. Also, we examined the correlations between mean LPP amplitudes at each electrode site and measures of self-efficacy and interpersonal relationships. We hypothesized that individuals with IGD would exhibit higher LPP amplitudes in response to game-related cues and that these amplitudes would be negatively correlated with levels of self-efficacy and interpersonal relationships.

## 2 Material and methods

### 2.1 Participants

**Figure 1:**
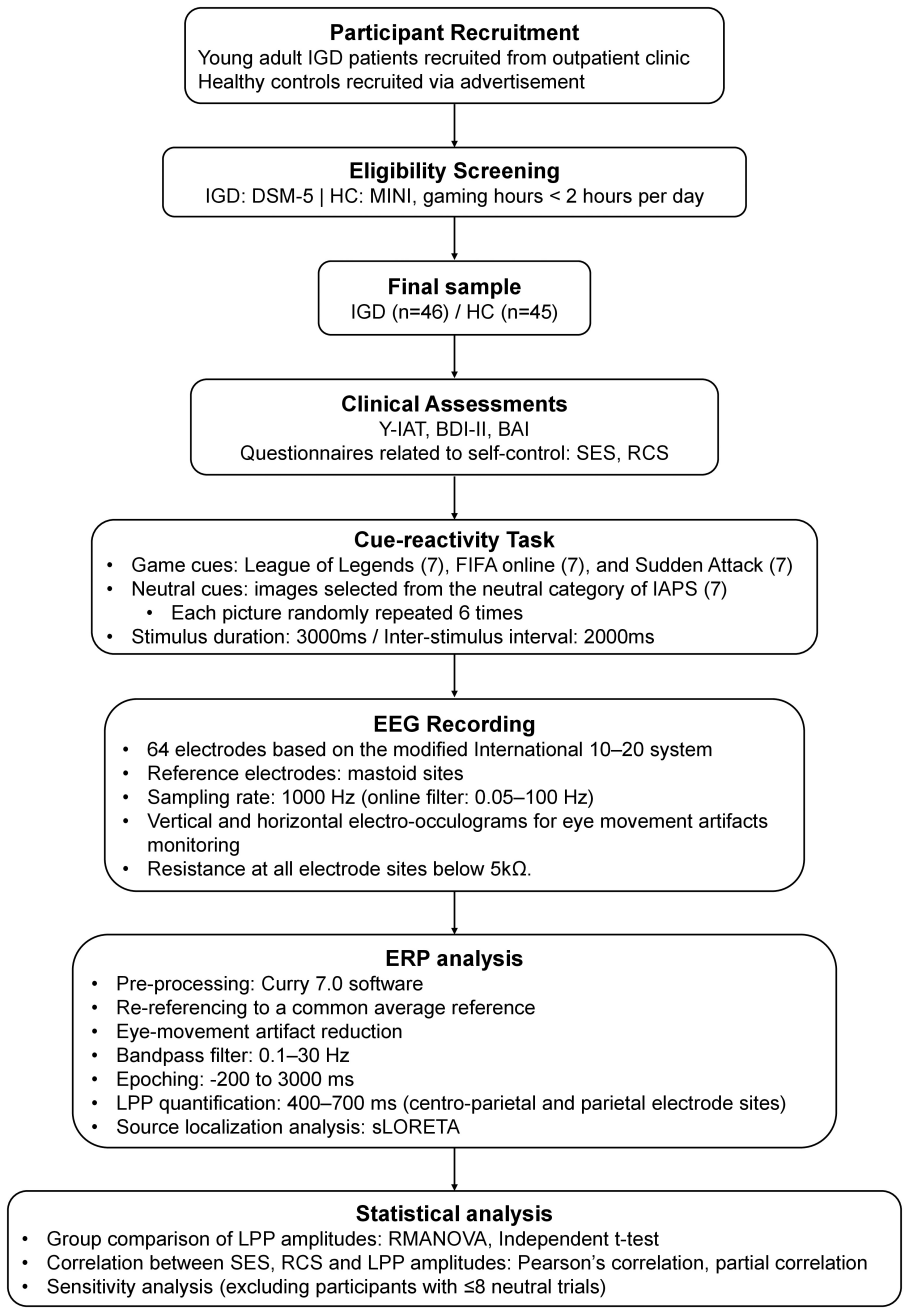
Overview of the experimental procedure. IGD, internet gaming disorder; HC, healthy control; DSM-5, diagnostic and statistical manual of mental disorders, fifth edition; MINI, mini-international neuropsychiatric interview; Y-IAT, young's internet addiction test; BDI, beck's depression inventory; BAI, beck's anxiety inventory; SES, generalized self-efficacy scale; RCS, relationship change scale; LPP, late positive potential.

The overall experimental procedure is illustrated in Figure 1. We recruited adult patients with IGD from the addiction outpatient clinic at the SMG-SNU Boramae Medical Center in Seoul, South Korea, and HC via an internet advertisement. A total of 91 participants were recruited, and 46 participants were diagnosed as IGD and the other 45 participants as healthy control (HC). Participants were aged 18–42 years in the IGD group and 19–32 years in the HC group. For each participant, the diagnosis of IGD was confirmed using the DSM-5 criteria through the interview conducted by an experienced psychiatrist. The HC participants were verified to have no current or lifetime psychiatric comorbidities, as assessed by the Mini-International Neuropsychiatric Interview, and engaged in internet gaming for less than 2 h per day across both weekdays and weekends. Gaming duration was assessed by self-report, and 2-h threshold was operationally defined to exclude individuals with potentially excessive gaming patterns (30). Patients with a lifetime diagnosis of substance abuse or dependence except for nicotine, neurological disorders, head trauma with loss of consciousness, and any medical illness with cognitive or sensory impairment, and intellectual disability were excluded from the study.

A subset of the current participants (*n* = 71) overlapped with those included in our previous study (16). The previous study analyzed 79 participants, whereas the present study includes 91 participants. The difference in sample size reflects differences in analytic eligibility (e.g., availability of self-efficacy measures) as well as the inclusion of newly recruited participants to address the current research question focusing on associations between LPP amplitudes and self-efficacy.

All the procedures of the research were adequately explained, and all participants gave their written informed consent. This study was conducted following the Declaration of Helsinki and was approved by the Institutional Review Board of SMG-SNU Boramae Medical Center, Seoul, Republic of Korea (IRB No. 01-2018-26).

### 2.2 Clinical assessments

All participants completed the Young's Internet Addiction Test (Y-IAT), the Beck Depression Inventory-II (BDI-II), and the Beck Anxiety Inventory (BAI) to assess the severity of IGD, depression, and anxiety, respectively. In addition, levels of self-efficacy and interpersonal relationships were assessed using the Generalized Self-Efficacy Scale (SES) and the Relationship Change Scale (RCS), respectively.

#### 2.2.1 Young's internet addiction test (Y-IAT)

The severity of IGD was assessed using a validated Korean version of the Y-IAT (31), which consists of 20 items rated on a 5-point Likert scale, yielding total scores ranging from 20 to 100. The Y-IAT has demonstrated good reliability in previous studies (31, 32).

#### 2.2.2 Beck depression inventory-II (BDI-II) and beck anxiety inventory (BAI)

Depressive and anxiety symptoms were assessed using the BDI-II (33) and the BAI (34), respectively. Both inventories consist of 21 items, with each item scored on a scale ranging from 0 to 3 according to symptom severity. These instruments have been widely validated and are commonly used as screening tools for depressive and anxiety symptoms (35).

#### 2.2.3 Generalized self-efficacy scale (SES)

The SES is a self-report questionnaire comprising 10 items, with responses rated on a 4-point scale ranging from 1 to 4. Higher scores indicate greater levels of self-efficacy (36). Self-efficacy refers to an individual's general confidence in their ability to cope effectively with challenging situations (37).

#### 2.2.4 Relationship change scale (RCS)

The RCS is a self-report questionnaire originally consisting of 27 items rated on a 5-point scale, with scores ranging from 1 to 5 (38). The scale was translated into Korean by Mun (1980) and subsequently revised to a 25-item questionnaire to better reflect Korean cultural contexts (39). In the revised version, total scores range from 25 to 125, with higher scores indicating more positive interpersonal relationships. The revised Korean version of the RCS was used in the present study.

### 2.3 EEG measurements

#### 2.3.1 Cue-reactivity task and EEG recording

In cue-reactivity task, all participants were given two groups of picture sets while recording their EEG. Participants did not provide subjective craving or urge ratings for individual stimuli during the cue-reactivity task. One group consisted of the game-related cues, including in-game screenshots from three popular internet games (i.e., League of Legends, FIFA online, and Sudden Attack). These three online games were selected because they were among the most widely played online games in Korea. All participants in the IGD group actively played all three games. The other group consisted of neutral images selected from the neutral category of the International Affective Picture System (IAPS) (40). Each category (League of Legends, FIFA online, Sudden Attack, and Neutral) included seven different pictures and the pictures were randomly repeated six times during the task. All visual pictures were standardized; 1,024 × 768 pixels of resolution, 361 × 271 of size, 72 dpi. The brightness, luminance, and color were also matched for each picture. Each picture was shown for 3,000 ms and the inter-stimulus interval was 2,000 ms during which an empty black screen was given. More detailed information about cue-reactivity task can be found in the previous report (16).

**Figure 2:**
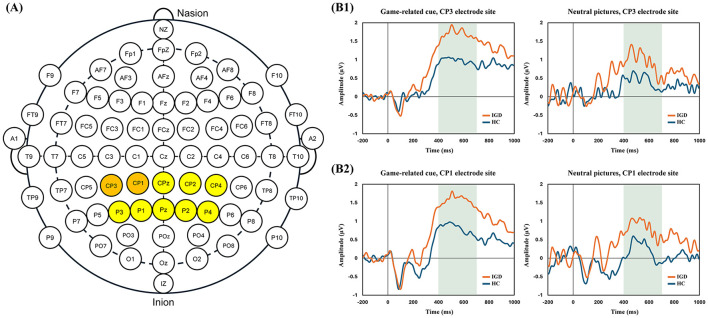
Grand-averaged late positive potential (LPP) waveforms. **(A)** Modified 10–20 electrode placement system. The centro-parietal (CP) and parietal (*P*) electrode sites used for analysis in the present study are highlighted. **(B)** Grand-averaged LPP waveforms in response to game-related cues and neutral pictures. The shaded area represents the time window used for LPP quantification (400–700 ms). **(B1)** Waveforms at the CP3 electrode site. **(B2)** Waveforms at the CP1 electrode site.

During the cue-reactivity task, continuous EEG recording based on the modified 10–20 international system (see Figure 2A) was conducted using a Neuroscan 64-channel Synamps system with a 64-channel Quick-Cap (Compumedics, Charlotte, NC, USA). The reference electrodes were placed at bilateral mastoid sites. The EEG recording was conducted at a 1,000 Hz sampling rate, and online filter of 0.05–100 Hz was applied. Electrode impedance was maintained below 5 kΩ at all recording sites throughout the task. For monitoring eye-movement artifacts, the vertical and horizontal electro-oculograms were recorded by electrodes of the left eye.

#### 2.3.2 ERP analysis

Pre-processing of the ERP data were performed using curry 7.0 software (Compumedics, Charlotte, NC, USA). The EEG data were re-referenced to a common average reference, and ocular artifacts were reduced using the artifact reduction algorithm in the Curry software (41). Eye blinks and eye movements were corrected based on the ocular artifact reduction method developed by Semlitsch et al. (41). Continuous EEG signals were recorded with a bandpass filter of 0.1–30 Hz and subsequently segmented into epochs from 200 ms pre-stimulus to 3,000 ms post-stimulus. Baseline correction was performed using the pre-stimulus interval voltage. Epochs containing EEG amplitudes exceeding ± 100 μV were automatically rejected. For comparison of EEG response to each type of stimuli, the epochs were sorted and averaged according to the stimulus type (game-related vs. neutral). The LPP were quantified as the mean values of amplitudes within the 400–700 ms time window at centro-parietal (CP3, CP1, CPz, CP2, and CP4) and parietal (P3, P1, Pz, P2, and P4) electrode sites (42, 43).

#### 2.3.3 Source localization of the ERP activity

Since EEG signals are recorded from superficial electrodes, inverse problem-solving procedure is required for the estimation of the source of electrophysiological activity. In this study, standardized low-resolution brain electromagnetic tomography (sLORETA) was employed for source localization, as it is one of the most widely used methods for estimating the source of neural activity in the brain (44, 45). In sLORETA, it is assumed that the source activation of a given voxel is synchronized with that of neighboring voxels for the calculation of a particular solution. A realistic head model based on the Montreal Neurological Institute 154 standard template was used for source estimation. In the template, 6,238 voxels with 5 × 5 × 5 mm resolution are provided, which are restricted to the cortical gray matter. For ERP peak and mean amplitude analyses in both groups, the same time window (400–700 ms) used for LPP calculation was applied. Source localization analyses were conducted using the absolute cue condition (i.e., game-related cues only), without computing a cue-minus-neutral contrast. All source localization analyses were conducted using the freely available sLORETA software (http://www.uzh.ch/keyinst/loretaOldy.htm).

For group comparisons, voxelwise statistical analyses were conducted using Statistical non-Parametric Mapping (SnPM) implemented in sLORETA. Independent-group comparisons were performed within the predefined time window (400–700 ms). Statistical inference was based on non-parametric permutation testing with 5,000 randomizations. Correction for multiple comparisons was performed at the voxel level using permutation-based family-wise error (FWE) control, and corrected critical thresholds and *p*-values were computed accordingly. Log-transformed ratios of averaged current density values were used for statistical estimation.

### 2.4 Statistical analysis

Demographic and clinical assessments were compared between the IGD group and HC using independent sample *t*-test. The χ^2^-test was used for categorical data analysis. Repeated-measures analysis of variance (RMANOVA) was performed for group comparisons of the mean LPP amplitudes, with two stimuli (game, neutral) and 10 centro-parietal electrode sites (CP3, CP1, CPz, CP2, CP4, P3, P1, Pz, P2, and P4) as the within-subject factors and the groups (IGD and HC) as between-subject factors. When the assumption of sphericity was violated, Greenhouse–Geisser correction was applied. Group comparison of the mean LPP amplitudes at each electrode was performed by independent *t*-test. Pearson's correlation analysis was performed to assess the association between the mean LPP amplitudes and SES and RCS in each group. The covariate was BDI and BAI for partial correlation analysis. A *p*-value < 0.05 was considered significant. To control for Type I error due to multiple testing, the false discovery rate (FDR) was controlled using the Benjamini–Hochberg procedure for electrode-wise comparisons and correlation analyses. All statistical analyses were conducted using SPSS software (version 27; IBM Corp., Armonk, NY, USA).

To examine the robustness of the findings, sensitivity analyses were conducted by excluding participants with eight or fewer accepted trials per condition (46).

## 3 Results

### 3.1 Demographic and clinical data

There were no significant group differences in sex and age, while the IGD group consumed significantly longer hours for internet gaming than the HC group (Table 1). On clinical assessments, the IGD group exhibited significantly higher scores on the Y-IAT, BDI-II, and BAI compared with the HC group. In contrast, scores on the SES, and RCS were significantly lower in the IGD group than in the HC group.

**Table 1:** Demographic and clinical characteristics of participants with internet gaming disorder and healthy controls.

Variables	Healthy control (*n* = 45) mean (*SD*)	Internet gaming disorder (*n* = 46) mean (*SD*)	χ^2^ or *t*	*p*-value	Effect size (Cramer's *V* for χ^2^; Cohen's d for *t*-test)
Demographic characteristics					
Sex (male/female)	(31/14)	(39/7)	3.238	0.072	0.19
Age (year)	24.78 (3.04)	24.74 (5.69)	−0.041	0.968	0.01
Time for internet gaming use on weekday (h/day)	0.23 (0.67)	5.28 (3.89)	8.582	< 0.001^***^	1.81
Time for internet gaming use on weekend day (h/day)	0.48 (1.12)	7.99 (9.65)	5.185	< 0.001^***^	1.09
Clinical assessments					
Y-IAT	29.67 (10.01)	60.11 (16.50)	10.641	< 0.001^***^	2.22
BDI	4.13 (4.19)	16.76 (10.99)	7.275	< 0.001^***^	1.51
BAI	3.44 (4.82)	13.72 (10.94)	5.818	< 0.001^***^	1.21
SES	30.31 (4.16)	24.52 (5.34)	−5.766	< 0.001^***^	1.21
RCS	94.09 (11.51)	76.33 (16.01)	−6.086	< 0.001^***^	1.27

Y-IAT, young's internet addiction test; BDI, beck's depression inventory; BAI, beck's anxiety inventory; SES, generalized self-efficacy scale; RCS, relationship change scale.

^*^P < 0.05; ^**^P < 0.01; ^***^P < 0.001.

### 3.2 LPP amplitudes

The mean number of accepted trials for the game-related condition was 55.70 (*SD* = 15.78, range 21–81) in the IGD group and 62.42 (*SD* = 14.73, range 17–83) in the HC group. For the neutral condition, the mean number of accepted trials was 16.91 (*SD* = 5.80, range 6–27) in the IGD group and 19.67 (*SD* = 5.53, range 8–28) in the HC group. Significant group differences were observed in the number of accepted trials for both the game-related and neutral conditions (*p* < 0.05).

In a full factorial RMANOVA including stimulus (game, neutral) and electrode as within-subject factors and group (IGD, HC) as a between-subject factor, a significant main effect of stimulus was observed [*F*_(1, 89)_ = 9.85, *p* = 0.002, partial η^2^ = 0.100], indicating larger LPP amplitudes for game-related stimuli relative to neutral. However, the Group × Stimulus interaction was not statistically significant [*F*_(1, 89)_ = 0.46, *p* = 0.499, partial η^2^ = 0.005], indicating that cue-specific reactivity did not significantly differ between groups. The Group × Stimulus × Electrode interaction was also not statistically significant [*F*_(4.24, 376.94)_ = 0.35, *p* = 0.855, partial η^2^ = 0.004].

To further explore potential regional differences, electrodes were grouped into centro-parietal (CP) and parietal (*P*) regions, and separate RMANOVAs were conducted. In the CP region, a significant Electrode × Group interaction was observed [*F*_(3.14, 279.70)_ = 4.13, *p* = 0.006, partial η^2^ = 0.044], suggesting differences in spatial distribution of LPP amplitudes across CP electrodes between groups. In contrast, Electrode × Group interaction was not significant in the *P* region. The Stimulus × Group interaction was not significant in both regions (all *p*-values > 0.05).

Exploratory comparisons at individual electrodes in CP region indicated nominal group differences at CP3 and CP1 (uncorrected *p* < 0.05; Table 2); however, these effects did not survive correction for multiple comparisons (Supplementary Table S11). Grand-averaged LPP waveforms of IGD and HC groups at the CP3 and CP1 electrode sites are shown in Figure 2B.

**Table 2:** Comparison of late positive potentials (LPPs) averaged between 400 ms and 700 ms post-stimulus onset between the internet gaming disorder and healthy control groups.

Variables	Healthy control (*n* = 45) mean (*SD*)	Internet gaming disorder (*n* = 46) mean (*SD*)	*T*	Uncorrected *p*-value	Effect size (Cohen's *d*)
Game stimuli					
CP3 electrode site	0.93 (1.65)	1.63 (1.58)	2.08	0.040^*^	0.44
CP1 electrode site	0.75 (1.48)	1.52 (1.83)	2.20	0.030^*^	0.46
CPz electrode site	0.59 (1.57)	0.99 (2.16)	1.00	0.320	0.21
CP2 electrode site	1.01 (1.59)	0.90 (2.31)	−0.27	0.787	0.06
CP4 electrode site	1.95 (1.42)	1.51 (1.78)	−1.31	0.194	0.28
Neutral stimuli					
CP3 electrode site	0.55 (1.68)	0.87 (2.13)	0.80	0.425	0.17
CP1 electrode site	0.38 (1.52)	0.81 (2.29)	1.06	0.294	0.22
CPz electrode site	0.46 (1.86)	0.48 (2.34)	0.05	0.964	0.01
CP2 electrode site	0.83 (1.88)	0.56 (2.50)	−0.59	0.556	0.12
CP4 electrode site	1.72 (1.72)	1.00 (1.93)	−1.88	0.064	0.39

^*^P < 0.05.

Complete RMANOVA results are provided in Supplementary Tables S5–7. Sensitivity analyses excluding participants with eight or fewer accepted trials per condition yielded comparable results (Supplementary Tables S8–10).

### 3.3 Correlation between LPP amplitudes and SES and RCS

**Figure 3:**
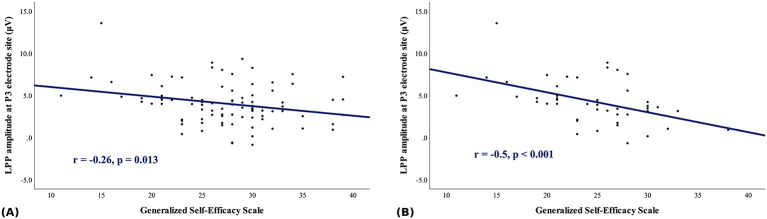
Correlation between generalized self-efficacy scale (SES) and late positive potential (LPP) amplitude at the P3 electrode site. **(A)** Correlation observed in the whole-sample. **(B)** Correlation observed in the Internet gaming disorder (IGD) group. (The *p* values displayed in the figure are uncorrected for multiple comparisons).

In the full-sample analysis, significant negative correlations were observed between SES scores and LPP amplitudes across parietal electrodes (P3, P1, Pz, P2, and P4) after adjusting for BDI and BAI (all FDR-corrected *p*-values < 0.05). In addition, nominal negative correlations were found between RCS scores and LPP amplitudes at several parietal electrodes (Pz, P2, and P4; uncorrected *p*-values < 0.05) after adjustment for BDI and BAI; however, these associations did not remain statistically significant after correction for multiple comparisons. In subgroup analysis of the IGD group, significant negative correlations were observed between SES scores and LPP amplitudes across parietal electrodes (P3, P1, Pz, P2, and P4), which remained statistically significant after adjustment for BDI, BAI and after FDR correction. In the HC group, no significant correlations were observed between the LPP amplitude and SES or RCS scores. For illustrative purposes, the partial correlation between SES scores and LPP amplitude at P3 (adjusted for BDI and BAI) is presented in Figure 3.

Complete correlation results across all electrodes and FDR-adjusted *p*-values are provided in Supplementary Tables S1, S2. Sensitivity analyses excluding participants with eight or fewer accepted trials per condition yielded comparable results (see Supplementary Tables S3, S4).

### 3.4 Source localization

**Figure 4:**
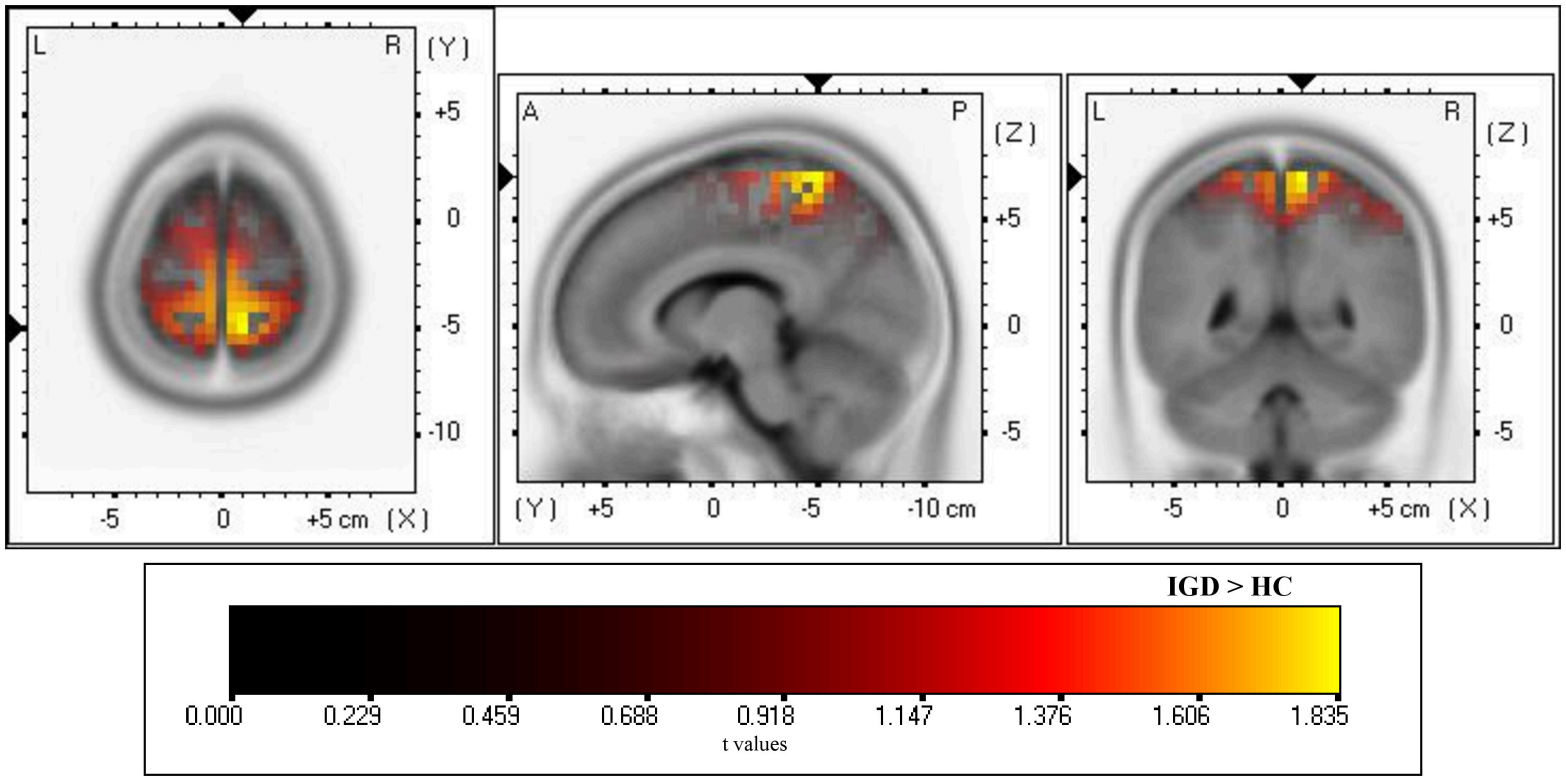
Source localization comparing the estimated current density of the internet gaming disorder (IGD) and healthy control (HC) groups for the game-related cues in the LPP time range (400–700 ms). Statistical maps were generated using SnPM (5,000 permutation randomizations) with voxel-level FWE correction for multiple comparisons. Significant voxels survived permutation-based correction at *p* < 0.05. A, anterior; P, posterior; L, left; R, right.

A significant group difference was observed in estimated current density at postcentral gyrus of parietal lobe (Brodmann area 5) in response to game-related cues (Figure 4). Specifically, the IGD group exhibited significantly higher current density in this region compared with the HC group. No significant group differences in current density were found in other brain regions.

## 4 Discussion

In this study, we investigated the differences in the mean LPP amplitudes and indicators of self-efficacy and interpersonal relationships between patients with IGD and healthy controls. To our knowledge, this is the first attempt to examine the potential association between LPP amplitudes and self-efficacy in the field of behavioral addictions. In clinical assessments with self-reports, patients with IGD exhibited higher levels of depression, anxiety and longer gaming hours compared to the HC group, which is consistent with previous studies (47, 48). In particular, the IGD group exhibited significantly lower levels of self-efficacy and interpersonal relationships, which were also consistent with previous studies (18, 19, 21). A significant main effect of stimulus was observed in the full factorial RMANOVA, indicating larger LPP amplitudes for game-related stimuli compared with neutral stimuli. Although no significant Group × Stimulus interaction was observed in the full factorial RMANOVA, a significant Group × Electrode interaction was detected within the centro-parietal region. Exploratory comparisons at individual electrodes suggested nominal group differences at CP1 and CP3, with higher LPP amplitudes in the IGD group. Although these differences did not remain significant after correction for multiple comparisons, small-to-moderate effect sizes were observed at these sites (Cohen's *d* = 0.44–0.46). This pattern may reflect an altered topographical pattern of resource allocation during cue processing in IGD, rather than a simple global increase in amplitude. Notably, there was a significant correlation between the level of self-efficacy and LPP amplitudes across parietal electrodes (P3, P1, Pz, P2 and P4), both before and after adjusting for the level of mood symptoms (BDI, BAI).

As implied by its definition (20), self-efficacy is closely related to one's belief in executive capacity to achieve specific accomplishments. Considering that self-efficacy has been proposed as a psychological factor associated with IGD (21), our finding of some correlations between self-efficacy level and LPP amplitude might provide additional insight into the neurophysiological correlates of IGD. Since higher LPP amplitude reflects greater motivational relevance to game-related cues, these results suggest that variability in self-efficacy levels is related to neurophysiological reactivity to game cues in IGD patients. However, given the correlational nature of our analyses, the directionality of this relationship cannot be determined.

It should be noted that the electrodes showing exploratory group difference in mean LPP amplitudes (i.e., CP3, CP1) and those showing correlation with self-efficacy level (i.e., parietal electrodes) were not identical. These findings do not suggest a uniform effect across the centro-parietal region. Rather, they may reflect two analytically distinct phenomena; categorical group differences between IGD and HC, and individual variability in self-efficacy within IGD participants. Since this is the first report of the potential correlation between LPP amplitudes and self-efficacy to our knowledge, further investigation is needed to clarify the spatial and functional organization of this relationship.

In source localization analysis, the IGD group exhibited significantly higher estimated current density in the postcentral gyrus in response to game-related cues, when compared to the HC group. This finding suggests that the scalp-level differences observed at centro-parietal electrodes may reflect underlying neural activity within parietal cortical regions. As the postcentral gyrus is linked to the somatosensory system, hyperactivation of this region might reflect enhanced sensorimotor processing to the game-related cues. Although the primary visual cortex is located in the occipital lobe, the superior parietal cortex has been reported to play a crucial role in visuo-motor integration process (49, 50). Moreover, sensorimotor integration is closely related to the formation of habitual behaviors (51). Therefore, hyperactivation of parietal regions observed in this study might be related to habit formation in response to the game-related visual stimuli. Considering that self-efficacy was negatively associated with LPP amplitudes, lower self-efficacy may be related to stronger cue-related processing. This interpretation is also consistent with the framework of motivational regulation described earlier. However, the relationship among self-efficacy, habit formation, and parietal activation remains to be clarified in future studies.

On the other hand, this finding from source localization analysis differs from our previous study, in which significantly higher estimated current density was observed in the superior and middle temporal gyri in the IGD group (16). This discrepancy can be explained in several ways. First, since sLORETA is based on a standardized head model, EEG source estimates may be influenced by various data-level factors, including individual anatomical variability. Second, different source localization results may reflect engagement of different brain regions within the same neural pathway, or different addictive pathways involved in IGD. This assumption is consistent with the inherent characteristics of EEG, which has high temporal resolution and therefore is well suited for spatially distributed and temporally dynamic patterns of neural activation in the brain (52).

Although this study yielded some meaningful findings, it has several limitations. First, we were unable to assess the gender differences of the result due to insufficient number of female participants. Since IGD is relatively less prevalent among females (53), future studies with larger samples with careful recruitment strategies would be needed. Second, because this study examined only the correlation between LPP amplitudes and self-efficacy levels, no conclusions can be drawn regarding the direction of causality. Future studies with formal moderation and mediation models are needed to clarify the potential causal or mechanistic relationships. Third, variability in accepted trial numbers across participants may have influenced signal precision. The IGD group showed fewer accepted trials per condition compared to the HC group. Although sensitivity analyses suggested that the main findings were robust, the potential influence of unequal trial numbers cannot be entirely excluded. In addition, the overall proportion of game-related to neutral stimuli was 3:1, as three game categories and one neutral category were included. This imbalance may have introduced differential habituation or salience effects across conditions, which should be considered when interpreting cue-reactivity findings. Fourth, subjective craving ratings were not collected during the cue-reactivity task, which limits direct interpretation of LPP responses in relation to conscious urge. Fifth, this study investigated only cross-sectional data assessing group differences. For more grounded evidence, longitudinal studies incorporating intra-individual analyses may be needed to determine whether changes in self-efficacy level are also associated with changes in LPP amplitudes within individuals over time. Finally, although sex and age were included as basic demographic variables, other factors, such as socio-economic status, might have confounded the results.

## 5 Conclusions

In conclusion, this study demonstrated that adults with IGD exhibited generally lower levels of self-efficacy and differences in the spatial distribution of LPP amplitudes in response to game-related cues in the centro-parietal regions. Importantly, a significant association was observed between LPP amplitudes in the parietal region and self-efficacy levels among individuals with IGD, which remained significant after adjusting for depressive and anxiety symptoms. Taken together, these findings suggest that LPP amplitudes may serve as a preliminary neurophysiological correlate associated with individual levels of self-efficacy among adults with IGD. However, replication as well as longitudinal studies are required to determine whether LPP amplitudes have potential utility as a clinically meaningful marker.

## Data Availability

The raw data supporting the conclusions of this article will be made available by the authors, without undue reservation.
