# Supplementary material for: Neurophysiological indicators of self-efficacy in internet gaming disorder: evidence from late positive potentials
Source: Front Public Health. 2026 Apr 14;14:1785973. doi: 10.3389/fpubh.2026.1785973 (PMC13121382; doi:10.3389/fpubh.2026.1785973)
Supplement: Supplementary file 1 [file Data_Sheet_1.docx]

**Supplementary Materials**

**Section S1. Correlation analyses between LPP amplitudes and self-efficacy measures**

**S1.1 Primary correlation analyses (whole sample)**

Table S1. Correlation matrix between LPP amplitudes and self-efficacy scale in the whole sample (N=91)

| **Electrode** | **r (SES)** | **p (SES)** | **FDR-corrected p (SES)** | **r (RCS)** | **p (RCS)** | **FDR-corrected p (RCS)** |
| --- | --- | --- | --- | --- | --- | --- |
| **CP3** | -0.07 | 0.540 | 0.675 | -0.07 | 0.503 | 0.559 |
| **CP1** | -0.13 | 0.231 | 0.385 | -0.17 | 0.108 | 0.170 |
| **CPz** | -0.03 | 0.812 | 0.899 | -0.04 | 0.690 | 0.690 |
| **CP2** | -0.11 | 0.298 | 0.426 | -0.17 | 0.119 | 0.170 |
| **CP4** | -0.01 | 0.899 | 0.899 | -0.13 | 0.221 | 0.276 |
| **P3** | -0.26 | 0.013 | 0.033* | -0.21 | 0.052 | 0.130 |
| **P1** | -0.24 | 0.021 | 0.042* | -0.17 | 0.107 | 0.170 |
| **Pz** | -0.28 | 0.007 | 0.033* | -0.24 | 0.025 | 0.125 |
| **P2** | -0.27 | 0.012 | 0.033* | -0.25 | 0.018 | 0.125 |
| **P4** | -0.27 | 0.009 | 0.033* | -0.22 | 0.041 | 0.130 |

Significant p-values after false discovery rate (FDR) correction are indicated as follows:
* p < 0.05, ** p < 0.01, *** p < 0.001.

Table S2. Correlation matrix between LPP amplitudes and self-efficacy scale in the IGD subgroup (N=46)

| **Electrode** | **r (SES)** | **p (SES)** | **FDR-corrected p (SES)** | **r (RCS)** | **p (RCS)** | **FDR-corrected p (RCS)** |
| --- | --- | --- | --- | --- | --- | --- |
| **CP3** | -0.26 | 0.089 | 0.111 | -0.02 | 0.906 | 0.906 |
| **CP1** | -0.29 | 0.059 | 0.090 | -0.20 | 0.202 | 0.253 |
| **CPz** | -0.12 | 0.445 | 0.445 | -0.06 | 0.701 | 0.779 |
| **CP2** | -0.28 | 0.063 | 0.090 | -0.30 | 0.046 | 0.115 |
| **CP4** | -0.15 | 0.320 | 0.356 | -0.22 | 0.148 | 0.211 |
| **P3** | -0.50 | < 0.001 | 0.003** | -0.27 | 0.075 | 0.150 |
| **P1** | -0.46 | 0.002 | 0.004** | -0.23 | 0.135 | 0.211 |
| **Pz** | -0.47 | 0.001 | 0.003** | -0.31 | 0.043 | 0.115 |
| **P2** | -0.47 | 0.001 | 0.003** | -0.34 | 0.022 | 0.110 |
| **P4** | -0.50 | < 0.001 | 0.003** | -0.36 | 0.017 | 0.110 |

Significant p-values after false discovery rate (FDR) correction are indicated as follows:
* p < 0.05, ** p < 0.01, *** p < 0.001.

**S1.2 Sensitivity correlation analyses (excluding participants with ≤ 8 neutral trials)**

Table S3. Correlation matrix for the sensitivity sample (N=85)

| **Electrode** | **r (SES)** | **p (SES)** | **FDR-corrected p (SES)** | **r (RCS)** | **p (RCS)** | **FDR-corrected p (RCS)** |
| --- | --- | --- | --- | --- | --- | --- |
| **CP3** | -0.07 | 0.551 | 0.689 | -0.07 | 0.525 | 0.583 |
| **CP1** | -0.14 | 0.216 | 0.337 | -0.18 | 0.106 | 0.212 |
| **CPz** | -0.02 | 0.833 | 0.833 | -0.05 | 0.685 | 0.685 |
| **CP2** | -0.13 | 0.236 | 0.337 | -0.15 | 0.181 | 0.259 |
| **CP4** | -0.03 | 0.825 | 0.833 | -0.12 | 0.265 | 0.331 |
| **P3** | -0.27 | 0.014 | 0.035* | -0.20 | 0.076 | 0.190 |
| **P1** | -0.25 | 0.024 | 0.048* | -0.17 | 0.133 | 0.222 |
| **Pz** | -0.29 | 0.009 | 0.035* | -0.24 | 0.030 | 0.150 |
| **P2** | -0.27 | 0.014 | 0.035* | -0.25 | 0.025 | 0.150 |
| **P4** | -0.28 | 0.010 | 0.035* | -0.21 | 0.053 | 0.177 |

Significant p-values after false discovery rate (FDR) correction are indicated as follows:
* p < 0.05, ** p < 0.01, *** p < 0.001.

Table S4. Correlation matrix for the IGD subgroup in the sensitivity sample (N=42)

| **Electrode** | **r (SES)** | **p (SES)** | **FDR-corrected p (SES)** | **r (RCS)** | **p (RCS)** | **FDR-corrected p (RCS)** |
| --- | --- | --- | --- | --- | --- | --- |
| **CP3** | -0.30 | 0.065 | 0.081 | -0.03 | 0.862 | 0.862 |
| **CP1** | -0.33 | 0.041 | 0.059 | -0.22 | 0.183 | 0.229 |
| **CPz** | -0.14 | 0.381 | 0.381 | -0.08 | 0.608 | 0.676 |
| **CP2** | -0.33 | 0.039 | 0.059 | -0.28 | 0.077 | 0.193 |
| **CP4** | -0.18 | 0.257 | 0.286 | -0.22 | 0.175 | 0.229 |
| **P3** | -0.53 | < 0.001 | 0.002** | -0.26 | 0.103 | 0.206 |
| **P1** | -0.50 | 0.001 | 0.002** | -0.24 | 0.135 | 0.225 |
| **Pz** | -0.50 | < 0.001 | 0.002** | -0.32 | 0.042 | 0.140 |
| **P2** | -0.50 | < 0.001 | 0.002** | -0.36 | 0.022 | 0.110 |
| **P4** | -0.55 | < 0.001 | 0.002** | -0.39 | 0.014 | 0.110 |

Significant p-values after false discovery rate (FDR) correction are indicated as follows:
* p < 0.05, ** p < 0.01, *** p < 0.001.

**Section S2. RMANOVA results**

* Stimulus (game, neutral) and Electrode (CP3, CP1, CPz, CP2, CP4, P3, P1, Pz, P2, P4) were treated as within-subject factors, and Group (IGD vs. HC) as a between-subject factor.

* Greenhouse–Geisser corrected degrees of freedom and p-values are reported when the assumption of sphericity was violated.

**S2.1 Whole-sample analyses**

Table S5. Full factorial RMANOVA results (all electrodes)

| **Effect** | **F** | **df1** | **df2** | **p-value** | **partial η²** |
| --- | --- | --- | --- | --- | --- |
| **Stimulus** | 9.85 | 1 | 89 | **0.002** | 0.100 |
| **Group** | 0.67 | 1 | 89 | 0.417 | 0.007 |
| **Electrode** | 107.92 | 3.59 | 319.90 | **< 0.001** | 0.548 |
| **Stimulus × Group** | 0.46 | 1 | 89 | 0.499 | 0.005 |
| **Stimulus × Electrode** | 1.20 | 4.24 | 376.94 | 0.309 | 0.013 |
| **Group × Electrode** | 1.82 | 3.59 | 319.90 | 0.131 | 0.020 |
| **Stimulus × Group × Electrode** | 0.35 | 4.24 | 376.94 | 0.855 | 0.004 |

Table S6. RMANOVA results for centro-parietal electrodes

| **Effect** | **F** | **df1** | **df2** | **p-value** | **partial η²** |
| --- | --- | --- | --- | --- | --- |
| **Stimulus** | 7.82 | 1 | 89 | **0.006** | 0.081 |
| **Group** | 0.16 | 1 | 89 | 0.693 | 0.002 |
| **Electrode** | 8.08 | 3.14 | 279.70 | **< 0.001** | 0.083 |
| **Stimulus × Group** | 1.08 | 1 | 89 | 0.301 | 0.012 |
| **Stimulus × Electrode** | 1.36 | 2.64 | 235.27 | 0.259 | 0.015 |
| **Group × Electrode** | 4.13 | 3.14 | 279.70 | **0.006** | 0.044 |
| **Stimulus × Group × Electrode** | 0.15 | 2.64 | 235.27 | 0.908 | 0.002 |

Table S7. RMANOVA results for parietal electrodes

| **Effect** | **F** | **df1** | **df2** | **p-value** | **partial η²** |
| --- | --- | --- | --- | --- | --- |
| **Stimulus** | 10.09 | 1 | 89 | **0.002** | 0.102 |
| **Group** | 0.91 | 1 | 89 | 0.342 | 0.010 |
| **Electrode** | 30.61 | 2.27 | 201.85 | **< 0.001** | 0.256 |
| **Stimulus × Group** | 0.10 | 1 | 89 | 0.749 | 0.001 |
| **Stimulus × Electrode** | 0.60 | 2.43 | 215.82 | 0.583 | 0.007 |
| **Group × Electrode** | 0.25 | 2.27 | 201.85 | 0.803 | 0.003 |
| **Stimulus × Group × Electrode** | 0.27 | 2.43 | 215.82 | 0.806 | 0.003 |

**S2.2 Sensitivity analyses**

Table S8. Full factorial RMANOVA results (all electrodes)

| **Effect** | **F** | **df1** | **df2** | **p-value** | **partial η²** |
| --- | --- | --- | --- | --- | --- |
| **Stimulus** | 10.08 | 1 | 83 | **0.002** | 0.108 |
| **Group** | 0.82 | 1 | 83 | 0.368 | 0.010 |
| **Electrode** | 100.00 | 3.60 | 298.49 | **< 0.001** | 0.546 |
| **Stimulus × Group** | 0.12 | 1 | 83 | 0.727 | 0.001 |
| **Stimulus × Electrode** | 1.01 | 4.22 | 350.62 | 0.404 | 0.012 |
| **Group × Electrode** | 1.37 | 3.60 | 298.49 | 0.247 | 0.016 |
| **Stimulus × Group × Electrode** | 0.43 | 4.22 | 350.62 | 0.801 | 0.005 |

Table S9. RMANOVA results for centro-parietal electrodes

| **Effect** | **F** | **df1** | **df2** | **p-value** | **partial η²** |
| --- | --- | --- | --- | --- | --- |
| **Stimulus** | 8.83 | 1 | 83 | **0.004** | 0.096 |
| **Group** | 0.23 | 1 | 83 | 0.637 | 0.003 |
| **Electrode** | 7.76 | 3.16 | 262.01 | **< 0.001** | 0.086 |
| **Stimulus × Group** | 0.50 | 1 | 83 | 0.483 | 0.006 |
| **Stimulus × Electrode** | 1.45 | 2.61 | 216.78 | 0.233 | 0.017 |
| **Group × Electrode** | 2.63 | 3.16 | 262.01 | **0.047** | 0.031 |
| **Stimulus × Group × Electrode** | 0.33 | 2.61 | 216.78 | 0.777 | 0.004 |

Table S10. RMANOVA results for parietal electrodes

| **Effect** | **F** | **df1** | **df2** | **p-value** | **partial η²** |
| --- | --- | --- | --- | --- | --- |
| **Stimulus** | 9.47 | 1 | 83 | **0.003** | 0.102 |
| **Group** | 1.08 | 1 | 83 | 0.301 | 0.013 |
| **Electrode** | 28.88 | 2.22 | 184.42 | **< 0.001** | 0.258 |
| **Stimulus × Group** | 0.00 | 1 | 83 | 0.975 | 0.000 |
| **Stimulus × Electrode** | 0.43 | 2.49 | 206.98 | 0.696 | 0.005 |
| **Group × Electrode** | 0.20 | 2.22 | 184.42 | 0.843 | 0.002 |
| **Stimulus × Group × Electrode** | 0.30 | 2.49 | 206.98 | 0.791 | 0.004 |

**Section S3. Electrode-wise group comparisons**

Table S11. Electrode-wise group comparisons of LPP amplitudes for game-related cues

|  | **Healthy control (n=45)**  **Mean (SD)** | **Internet gaming disorder (n=46)**  **Mean (SD)** | **t** | **Uncorrected p-value** | **FDR corrected p-value** | **Effect size (Cohen’s d)** |
| --- | --- | --- | --- | --- | --- | --- |
| **Game stimuli** |  |  |  |  |  |  |
| **CP3 electrode site** | 0.93 (1.65) | 1.63 (1.58) | 2.08 | 0.040 | 0.100 | 0.44 |
| **CP1 electrode site** | 0.75 (1.48) | 1.52 (1.83) | 2.20 | 0.030 | 0.100 | 0.46 |
| **CPz electrode site** | 0.59 (1.57) | 0.99 (2.16) | 1.00 | 0.320 | 0.400 | 0.21 |
| **CP2 electrode site** | 1.01 (1.59) | 0.90 (2.31) | -0.27 | 0.787 | 0.787 | 0.06 |
| **CP4 electrode site** | 1.95 (1.42) | 1.51 (1.78) | -1.31 | 0.194 | 0.323 | 0.28 |
